# Automatic diagnosis of macular diseases from OCT volume based on its two-dimensional feature map and convolutional neural network with attention mechanism

**DOI:** 10.1117/1.JBO.25.9.096004

**Published:** 2020-09-16

**Authors:** Yankui Sun, Haoran Zhang, Xianlin Yao

**Affiliations:** Tsinghua University, Department of Computer Science and Technology, Beijing, China

**Keywords:** optical coherence tomography, convolutional neural network, transfer learning, image classification, attention mechanism

## Abstract

**Significance:** Automatic and accurate classification of three-dimensional (3-D) retinal optical coherence tomography (OCT) images is essential for assisting ophthalmologist in the diagnosis and grading of macular diseases. Therefore, more effective OCT volume classification for automatic recognition of macular diseases is needed.

**Aim:** For OCT volumes in which only OCT volume-level labels are known, OCT volume classifiers based on its global feature and deep learning are designed, validated, and compared with other methods.

**Approach:** We present a general framework to classify OCT volume for automatic recognizing macular diseases. The architecture of the framework consists of three modules: B-scan feature extractor, two-dimensional (2-D) feature map generation, and volume-level classifier. Our architecture could address OCT volume classification using two 2-D image machine learning classification algorithms. Specifically, a convolutional neural network (CNN) model is trained and used as a B-scan feature extractor to construct a 2-D feature map of an OCT volume and volume-level classifiers such as support vector machine and CNN with/without attention mechanism for 2-D feature maps are described.

**Results:** Our proposed methods are validated on the publicly available Duke dataset, which consists of 269 intermediate age-related macular degeneration (AMD) volumes and 115 normal volumes. Fivefold cross-validation was done, and average accuracy, sensitivity, and specificity of 98.17%, 99.26%, and 95.65%, respectively, are achieved. The experiments show that our methods outperform the state-of-the-art methods. Our methods are also validated on our private clinical OCT volume dataset, consisting of 448 AMD volumes and 462 diabetic macular edema volumes.

**Conclusions:** We present a general framework of OCT volume classification based on its 2-D feature map and CNN with attention mechanism and describe its implementation schemes. Our proposed methods could classify OCT volumes automatically and effectively with high accuracy, and they are a potential practical tool for screening of ophthalmic diseases from OCT volume.

## 1 Introduction

Macular diseases have received widespread attention in recent years, and age-related macular degeneration (AMD) and diabetic macular edema (DME) are two common diseases that cause severe vision loss and blindness, especially in adults. Optical coherence tomography (OCT) is an imaging technology that measures the backward scattered light intensity of objects.^1^ Since the structure of the retina can be clearly visualized with micron resolution using OCT, some eye diseases such as AMD and DME can be diagnosed based on OCT images.^2^^,^^3^ In clinical diagnosis, ophthalmologists make diagnostic decisions of retina edema diseases based mainly on the observation and analysis of OCT images. Spectral-domain OCT (SD-OCT) has been capable of generating 3D datasets since its inception and is widely used in clinics. One retinal OCT volume usually contains dozens or even hundreds of B-scans, and ophthalmologists need to manually identify retina lesions at each cross-section of the OCT volume and then make diagnostic decisions related to ocular diseases. This greatly increases the analysis burden of the eye specialist, and this manual interrogation requires expert graders, which is inefficient and prone to yielding subjective results. Consequently, high-performance automatic 3-D OCT image analysis is critical for the diagnosis of retinal disease.

A convolutional neural network (CNN or ConvNet) is one of the most popular algorithms for deep learning. It has an input layer, an output layer, and various hidden layers including convolutional layers, pooling layers, and other layers for processing. The CNN model can learn image features and train classifiers simultaneously from a large number of annotated images, by which a hierarchy of image features can be learned automatically. For CNN models developed based on natural images, their weights could be adjusted for the specific purpose of the intended work such as in OCT image analysis using knowledge transfer. To solve the OCT volume classification problem, a scheme to extract all B-scan feature vectors of an OCT volume is proposed based on transfer learning. They are then stacked together to obtain a two-dimensional (2-D) feature map of the OCT volume for classification. The proposed method has the advantage of improving automated analysis, yielding objective results, and increasing the accessibility of 3-D OCT images.

Over the past years, numerous automated macular OCT classification techniques have been developed, and they could be chiefly categorized into two types.

### 1.1 OCT Image Classification

This method focuses on generating 2-D OCT image (i.e., B-scans) classifiers when OCT images and their labels are provided. Traditional machine learning is often used in OCT image classification. These methods first extract B-scan features and subsequently design classifiers.^4^^–^^7^ Srinivasan et al.^5^ extracted histograms of oriented gradients features of B-scans and then classified them using a support vector machine (SVM) classifier. Sun et al.^6^^,^^7^ performed feature extraction of B-scans using dictionary learning, sparse coding, and spatial pyramid matching and recognized them using an SVM classifier. Another useful technique is deep learning, especially CNN classifier models. Several works on macular OCT image classification using CNN models have been conducted.^8^^–^^13^ The attention mechanism in deep learning is similar to the attention mechanism of human vision in that it focuses attention on important points among a large number of information, selecting key information and ignoring other unimportant information. In OCT image classification, attention could focus on lesion part, which usually occupies only a very small part of the OCT scan, and it has been explored and introduced for macular OCT classification applications.^14^^,^^15^

### 1.2 OCT Volume Classification

This method focuses on generating OCT volume (i.e., a series of B-scans) classifiers when OCT volumes and their volume-level labels are known. A voting inference strategy is often applied to OCT volume classification. For an OCT volume, the voting strategy involves initially obtaining all of its B-scan classifications, and then it yields a volume-level classification based on the results. Majority voting has been used in several studies.^5^^–^^7^^,^^12^ Rasti et al.^16^ presented a multiscale CNN ensemble structure and suggested a voting strategy to obtain volume-level diagnosis. Qiu et al.^17^ proposed a B-scan classifier using a relabeling technique and suggested another voting strategy to classify OCT volume. Another important technique is true volume-level OCT data classification.^18^^–^^29^ For an OCT volume, it first obtains a global feature representation of the volume and then designs classifiers to recognize it. Venhuizen et al.^20^^,^^21^ obtained the global representation of an OCT volume using clustering and bag-of-word models and classified it using a random forest classifier. Fang et al.^23^ extracted the global feature of an OCT volume based on the combination of principal component analysis network (PCANet)^22^ and composite kernels and recognized it using an extreme learning machine. Rasti et al.^24^ obtained the global feature of an OCT volume using a wavelet-based convolutional neural network and classified it using a random forest classifier. Apostolopoulos et al.^25^ directly tiled all of the B-scans in a volume vertically in a 2-D plane to obtain the global feature of an OCT volume and obtained its classification using a 2-D CNN classifier. De Fauw et al.^26^ obtained a tissue-segmentation map of an OCT volume as a global feature and classified it using a deep learning architecture. Santos et al.^27^ extracted the global feature of an OCT volume from the perspective of a C-scan using semivariogram and semimadogram functions and recognized it using an SVM. Seebock et al.^28^ obtained the feature representation of a retinal OCT volume using deep denoising autoencoders to segment anomalous regions and employed clustering to classify it. Sun et al.^29^ proposed multiple instances of a learning-based SVM to perform volumetric classification using features extracted from the histogram obtained from oriented gradient and principal component analysis.

In this report, we propose a method to extract the global feature of an OCT volume for OCT volume classification. Specifically, for an OCT volume, we extract its B-scan feature vectors and stack them together to generate a 2-D feature map that contains the global features of the OCT volume. The 2-D feature map is then used to recognize the OCT volume using a volume-level OCT volume classifier. We fine-tune a pretrained CNN classifier as a B-scan feature extractor based on transfer learning and train a volume-level OCT volume classifier using 2-D feature maps. Therefore, OCT volume classification is successfully implemented through two image classifiers. We design some volume-level classifiers for 2-D feature maps. In particular, we propose a classifier, i.e., convolutional neutral network with attention mechanism. To the best of our knowledge, this is the first algorithm to introduce the 2-D feature map of an OCT volume for classification.^30^ The main contributions of this report are described as follows:

• We propose a method to obtain a 2-D feature map of a retinal OCT volume.
• We propose a deep learning architecture for OCT volume classification based on 2-D feature representation and transfer learning and an effective CNN with attention mechanism classifier to classify 2-D feature maps.
• We implement OCT volume classification using two 2-D image classifiers: one is based on B-scans and the other is based on 2-D feature maps. This could address the storage and computation complexity problems associated with large scale OCT volume recognition applications.
• Without any OCT image preprocessing steps such as denoising and flattening, the proposed method achieves desirable volume classification results.

The remainder of this report is organized as follows: Sec. 2 describes our proposed methods. Experimental results on clinical OCT datasets and discussions are presented in Sec. 3, and Sec. 4 summarizes the main conclusions.

## 2 Methods

**Fig. 1:**
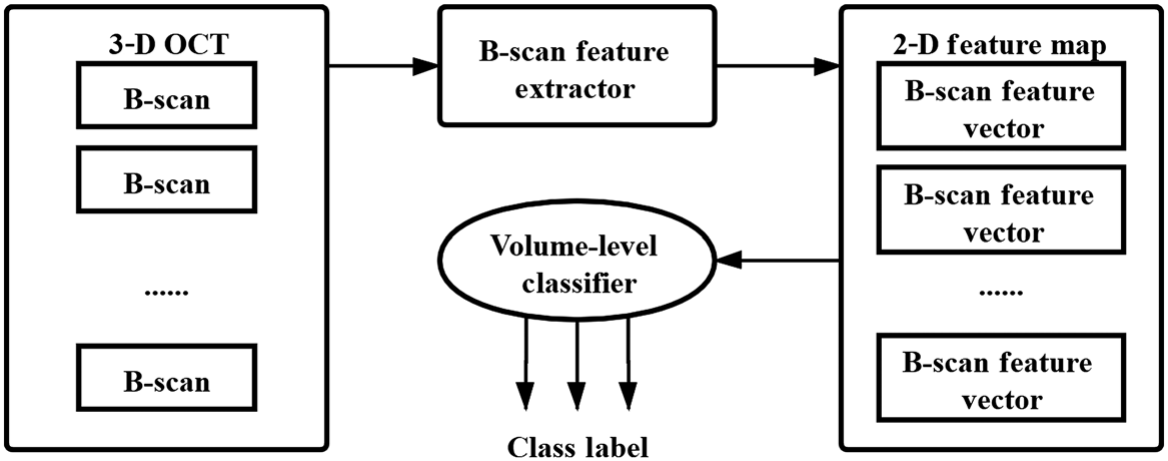
Architecture of our OCT volume classification.

We propose a general framework for OCT volume classification. The basic concept is to obtain the 2-D feature map of an OCT volume by extracting and stacking all of its B-scan feature vectors together. The 2-D feature map is then used for OCT volume classification. The architecture of our OCT volume classification is shown in Fig. 1.

Our proposed classification architecture consists of three modules:

(1) B-scan feature extractor. For an OCT volume, this module is responsible for extracting all of its B-scan feature vectors. B-scan features could be manually selected features, learned features, or their combinations;
(2) 2-D feature map. All of the B-scan feature vectors of the OCT volume are concatenated row-by-row to obtain a 2-D feature map as its global feature representation;
(3) Volume-level classifier. A 2-D feature map is used as its input to classify the OCT volume. In general, volume-level classifiers could be traditional machine learning methods such as Naive Bayes, NB; support vector machine, SVM; k-nearest neighbor, k-NN; and random forest, RF or deep learning methods such as CNN. We mainly focus on SVM and CNN in this report.

Here, we mainly describe a volume classification network based on CNN models and show how to train it. Specifically, for a given labeled OCT volume dataset, we divide it into a volume-level training set and a test set. We then show how to fine-tune a pretrained CNN model for B-scan feature extraction, obtain the 2-D feature map of an OCT volume, and train a volume-level CNN classifier based on the 2-D feature maps.

### 2.1 CNN Model for B-Scan Feature Extraction

#### 2.1.1 Pretrained ResNet-50

**Fig. 2:**
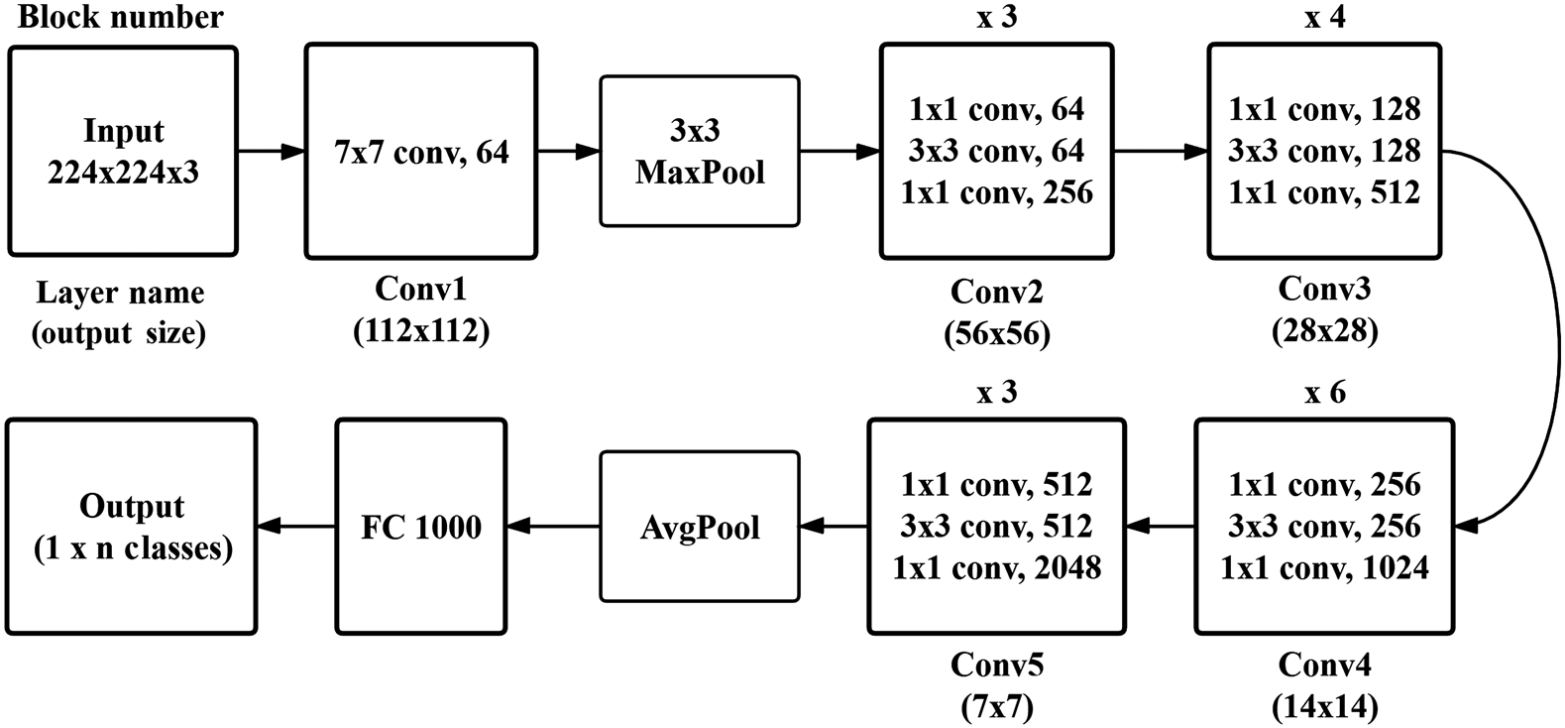
Network architecture of ResNet-50.

The CNN model has been widely used in computer vision (especially in image classification) since the ImageNet^31^ competition in 2012. In recent years, variants of CNN architectures such as AlexNet,^32^ VGG,^33^ GoogLeNet,^34^ and ResNet^35^ have been developed. In principle, all of these CNN models can be used to train our models based on transfer learning to adapt specific datasets such as an OCT image dataset. ResNet is a representative deep network. In this study, we take ResNet-50 as an example to show how to obtain a CNN model as a B-scan feature extractor. The architecture of ResNet-50 is shown in Fig. 2. The input of ResNet-50 is an image of size 224×224 with RGB channels, followed by convolutional building blocks: one Conv1, three Conv2, four Conv3, six Conv4, and three Conv5, and a feature vector of size 2048 is obtained using the AvgPool module on the output of the last Conv5 block as the input of a fully connected (FC) layer. “FC 1000” stands for an FC layer with 1000 class outputs.

The most straightforward approach for obtaining a CNN model for B-scan feature extraction is to utilize the pretrained ResNet-50 as a feature extractor. Specifically, we fix all of the pretrained weights in all of the Conv1 to Conv5 blocks and ignore the FC layer. For each B-scan of an OCT volume, we take the average pooling of the output of the Conv5 block as the B-scan feature vector. The advantage of this method is that no training of the ResNet-50 is required; therefore, it is efficient. Yet, because it does not exploit the advantages of the characteristics of OCT images, the extracted B-scan feature vector is often inadequate.

#### 2.1.2 Finetuned ResNet-50

A better approach is to fine-tune the pretrained ResNet-50 using the OCT volume dataset and to use it as a B-scan feature extractor. Fine-tuning ResNet-50 includes generating B-scan training samples and updating the weight parameters.

##### Generating B-scan training samples

In our OCT volume classification, labels of OCT volumes are given; they are AMD, DME, or NOR. However, labels of B-scans in an OCT volume are not known. Here, we simply assign the label of each volume to each B scan in the corresponding volume. All of the OCT B-scans in the volume-level training set constitute the B-scan training samples that are used to finetune ResNet-50 using transfer learning.

##### Finetuning the pretrained model

We transfer all of the pretrained weights in the Conv1 to Conv5 blocks of ResNet-50 and modify the FC layer to output a K class to suit our dataset, where K is the label number in the OCT volume dataset. The FC weights are generated randomly. We repeat the same B-scan (gray image) in the RGB channels as the input of ResNet-50 and retrain the specified weights in the Conv1 to Conv5 blocks and FC using the B-scan training samples.

### 2.2 2D Feature Map

For a given OCT volume $X_{i}$, we extract its B-scan feature vectors using a B-scan feature extractor and obtain its 2-D feature map by concatenating all of its B-scan feature vectors row-by-row. Suppose each B-scan feature vector is of size L, where L is far less than the B-scan’s dimension. In particular, if we use the finetuned ResNet-50 as the B-scan feature extractor, then we have L=2048. Evidently, the 2-D feature map of the volume $X_{i}$ is a 2-D matrix of size $\left(|X_{i}|,L\right)$, where $\left|X_{i}\right|$ is the number of B-scans of $X_{i}$. This global feature representation possesses the following advantages:

(1) Data dimension reduction.

In the public Duke dataset,^36^ each volume consists of 100 B-scans and each B-scan is an image of size 1000×512. Therefore, using its 2-D feature map representation, its dimensions could be reduced from 1000×100×512=51,200,000 to 100×2048=204,800.

(2) Data correlation.

The 2-D feature map of the OCT volume has data correlation between the rows, as illustrated in Sec. 3.5.3.

(3) From 3-D to 2-D.

Since image classifiers could be used on 2-D feature maps, this representation transforms OCT volume classification into image classification.

### 2.3 Volume-Level CNN Classifier with/without Attention Mechanism

**Fig. 3:**
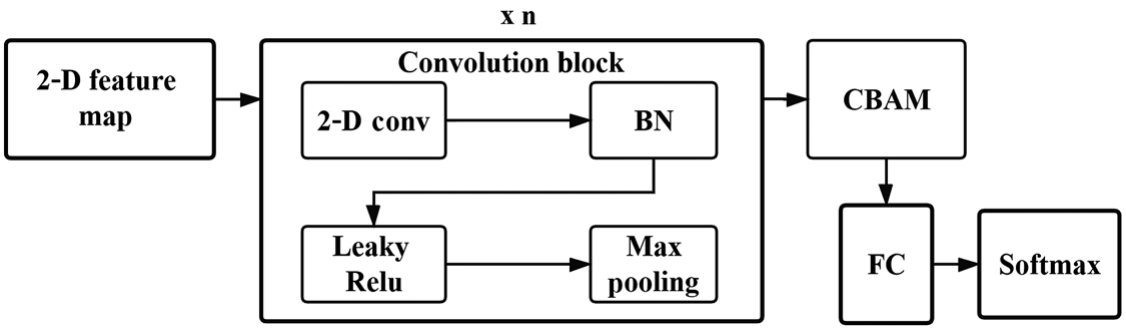
CNN classifier structure.

Various classifiers could be designed to classify 2-D feature maps. Although the 2-D feature map is different from natural image and OCT B-scan image, it also has data correlation. Therefore, a CNN image classifier could be designed to deal with it. Furthermore, convolution operations extract informative features by blending cross-channel and spatial information together, while the convolutional block attention module (CBAM)^37^ could be used to emphasize meaningful features along those two principal dimensions: channel and spatial axes. Specifically, given an intermediate feature map, CBAM sequentially infers attention maps along two separate dimensions: channel and spatial; then the attention maps are multiplied to the input feature map for adaptive feature refinement. Based on these ideas, we propose a volume-level classifier with attention mechanism, denoted by CNN_CBAM, as is shown in Fig. 3. This architecture consists of n convolution blocks, a CBAM module, an FC layer and a softmax classifier, where two to the power of n is less than the row number of the 2-D feature map. Each convolution block is a standard CNN module, including a 2-D convolution layer, batch normalization,^38^ Leaky ReLU, and max pooling. Each convolution block has a 3×3 kernel with four channels. In the case of n=4, for an input of a 2-D feature map with a size of 100×2048, the outputs of the four convolution blocks are 4×50×1024, 4×25×512, 4×12×256, and 4×6×128 sequentially; then the output 4×6×128 is refined in features by CBAM and transferred into a one-dimensional (1-D) vector of size 3072 as the input of the FC layer. We note that CNN_CBAM becomes CNN volume-level classifier when the CBAM module is ignored.

For any OCT volume in the volume-level training dataset, we first obtain its 2-D feature map using the finetuned ResNet-50 as the feature extractor and assign its label as the same as the label of the OCT volume. Then, we train the volume-level CNN or CNN_CBAM classifier model using all of the 2-D feature maps and their labels.

For any OCT volume in the volume-level test dataset, as shown in the flowchart in Fig. 1, we extract all of its B-scan feature vectors using the finetuned ResNet-50 model as a feature extractor and stack them together to obtain its 2-D feature map. Finally, we input the 2-D feature map in the trained CNN or CNN_CBAM volume-level classifier to recognize the OCT volume.

## 3 Experimental Results and Discussion

### 3.1 Experimental Environments

Our experiments were performed on a machine with an IntelCorei7-7700K 4.20 GHz CPU, 32 GB RAM, NVIDIA Titan X GPU, 12 GB RAM, and Windows 10 operating system. We use PyTorch as the deep learning framework, powered by the cuDNN Toolbox, and compiled the code in Python 3.6.

### 3.2 Datasets

One test dataset is a publicly available two-class dataset released by the VIP Lab of Duke University,^36^ in which the OCT volume data were acquired with a Bioptigen SD-OCT system. This dataset consists of 269 intermediate AMD volumes and 115 normal volumes, with each volume having ∼100 B-scans and each B-scan being of size 1000×512. The other dataset is from Tsinghua University. The OCT volume data were obtained with Spectralis (Heidelberg Engineering, Heidelberg, Germany) in Beijing Hospital. It consists of 448 AMD volumes and 462 DME volumes. Each volume has 25 B-scans, and each B-scan is of size 1000×512.

### 3.3 Experimental Settings

For all B-scans in OCT volumes, we standardize them with the mean value of 0.45 and the standard deviation of 0.23, as ResNet-50 does usually. During the training stage, we fix weight parameters in the Conv1 to Conv3 blocks and train weight parameters in the Conv4 and Conv5 models and in the FC layer, alternately using B-scan training samples. To prevent the model from overfitting, data augmentation techniques are used. Specifically, we adopt two data augmentation strategies on each B-scan in the training set: cropping and horizontally flipping. Cropping can increase the diversity of data samples when the proper cropping parameter is selected, and horizontally flipping can preserve the generalization of the left and right eye samples. In our experiments, we do data augmentation online during the training stage as follows: (1) make a crop of random size (0.7 to 1.0) of the original B-scan size and a random aspect ratio (3/4 to 4/3) of the original aspect ratio and then resize it to 224×224; (2) horizontally flip the cropped B-scan randomly with a 50% probability, i.e., randomly generate a number from 0 to 1 and flip the image if the number is <0.5.

Softmax cross-entropy loss and momentum-based stochastic gradient descent (SGD) are utilized to train the model. The learning rate is set to $5\times 10^{-4}$, and the momentum factor is set to 0.95. At each iteration, the minibatch size is set to 64 B-scans. The number of epochs is set to 3. As a volume-level classifier, the linear support vector machine (LSVM) with a $L_{2}$ penalty and squared hinge loss is used. We transfer the 2-D feature map of a volume into a 1-D feature vector with lexicographical order for the input of LSVM. The weight parameters of the CNN classifier as shown in Fig. 3 are initially trained using 2-D feature maps with a size of 100×2048 for the Duke dataset and 25×2048 for our private dataset. Softmax cross-entropy loss and momentum-based SGD are used to train the model. The learning rate is set to $1\times 10^{-3}$, and the momentum factor is set to 0.9. The number of epochs is set to 150, and the minibatch size is set to 64 2-D feature maps at each iteration. For the CNN_CBAM classifier, the code of CBAM is available at the Github repository: https://github.com/Jongchan/attention-module.

### 3.4 Evaluation Metrics

We utilize accuracy (ACC), sensitivity (SE), and specificity (SP) to evaluate the performance of our proposed methods. They are defined as

$$\mathrm{ACC}=\frac{\mathrm{TP}+\mathrm{TN}}{\mathrm{TP}+\mathrm{TN}+\mathrm{FP}+\mathrm{FN}},$$

$$\mathrm{SE}=\frac{\mathrm{TP}}{\mathrm{TP}+\mathrm{FN}},$$

$$\mathrm{SP}=\frac{\mathrm{TN}}{\mathrm{TN}+\mathrm{FP}},$$

where TP is the true positive, FN is the false negative, TN is the true negative, and FP is the false positive.

We perform one fivefold cross-validation for both datasets. Each class of volumes is randomly divided into five approximately equal subsets at the volume level. The experiments are repeated five times, and for each experiment, four subsets are used as the training set and the remaining set is used as the test set. We report the mean and standard deviation of the metrics ACC, SE, and SP for each method.

### 3.5 Experiments on Duke Dataset

In our experiments, all AMD volumes are randomly divided into five approximately equal parts, denoted as AMD_1, …, AMD_5. Similarly, normal volumes are divided into five parts as NOR_1, …, NOR_5. By doing so, we split the Duke dataset into five parts, D_i; here D_i consists of all of the OCT volumes in AMD_i and NOR_i, i=1,2,3,4,5. For each fold experiment, four parts of D_1, …, D_5 are used as the training set to finetune ResNet-50 and to train the volume-level classifier, and the remaining set is used as the test set to evaluate the volume-level classifier.

#### 3.5.1 Ablation studies

In this section, ablation studies are performed to investigate the effects of using different CNN models as B-scan feature extractors and volume-level classifiers on classification performances.

We test our implementation schemes using the pretrained ResNet-50, the finetuned ResNet-50 without data augmentation (FT-ResNet50), and the finetuned ResNet-50 with data augmentation (FTA-ResNet50) as B-scan feature extractors and SVM, CNN, and CNN_CBAM as volume-level classifiers. For convenience, we denote our classification methods simply. For example, FTA-ResNet50+CNN denotes that FTA-ResNet50 and CNN are used as the B-scan feature extractor and volume-level classifier, respectively. The experimental results of our proposed methods are demonstrated in Table 1, where NOR is negative, and AMD is positive.

**Table 1:** Performances of our proposed methods on two classes of the Duke dataset (%).

Methods	ACC	SE	SP
ResNet50+SVM	95.06±1.50	96.30±2.03	92.17±5.07
ResNet50+CNN	94.53±1.72	95.53±1.50	92.17±3.25
FT-ResNet50+SVM	96.09±0.82	99.62±0.75	87.83±3.25
FT-ResNet50+CNN	96.36±1.72	99.25±0.92	89.57±6.51
FTA-ResNet50+SVM	97.92±0.63	98.52±1.39	96.52±1.74
FTA-ResNet50+CNN	97.65±0.99	99.26±0.91	93.91±3.48
FTA-ResNet50+CNN_CBAM	98.17±0.64	99.26±0.91	95.65±2.75

It can be seen from Table 1 that, for a fixed volume-level classifier, the finetuned ResNet-50 with data augmentation outperforms the finetuned ResNet-50 without data augmentation, and the latter is better than the pretrained ResNet-50. This shows that our proposed finetuned ResNet-50 with data augmentation as the B-scan feature extractor is the best. For a fixed B-scan feature extractor, CNN_CBAM outperforms CNN significantly, and SVM is almost the same as CNN. This implies that the CBAM attention module is very helpful for improving classification performance. As a whole, FTA-ResNet50+CNN_CBAM is the best of all. In particular, its sensitivity is larger than 99%.

#### 3.5.2 Comparison with state-of-the-art methods

We compare our methods with several methods, such as that proposed by Santos et al.^27^ and the voting method of Qiu et al.,^17^ on the Duke dataset. Santos et al. obtained classification results with an SVM classifier using fivefold cross-validation with 100 repetitions. Qiu et al. obtained classification results using a voting strategy using fivefold cross-validation with five repetitions.

**Fig. 4:**
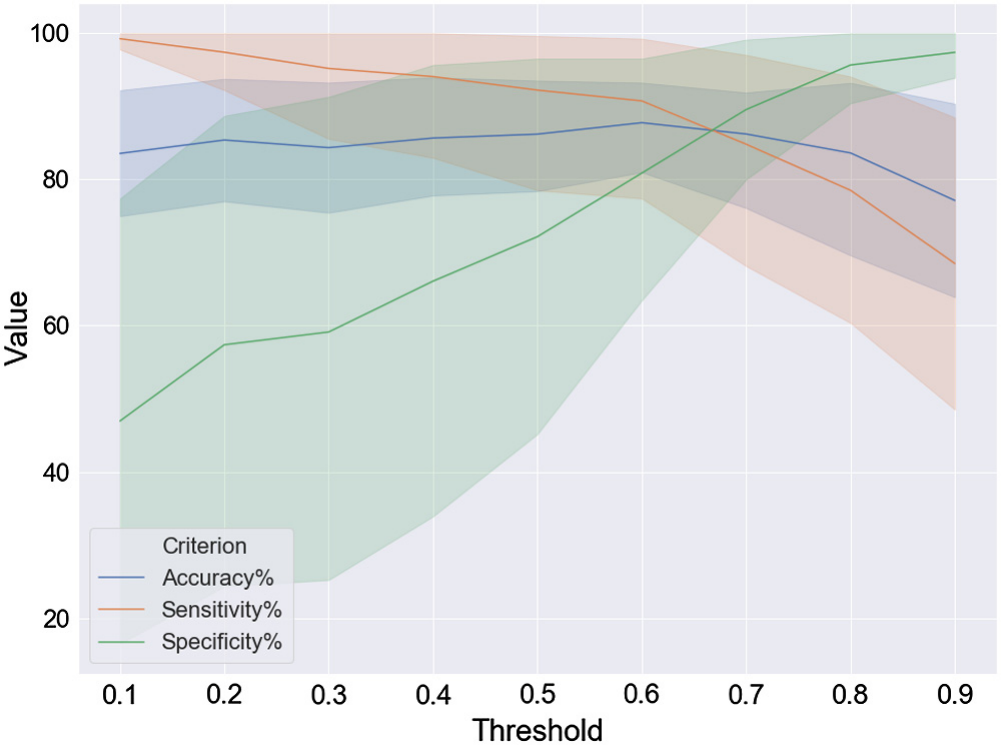
Voting results: the relationship between accuracy, sensitivity, specificity, and threshold.

In voting inference methods, we choose FTA-ResNet50 as the B-scan classifier. For any test OCT volume $X_{i}$, we perform classification on all of its B-scan $X_{ij}$ to get the class label $Y_{ij}$. Let ε be a threshold, according to a voting inference strategy, we obtain the volume-level label $Y_{i}$ of $X_{i}$ by computing $P_{i}^{L}$ and comparing it with ε, where $P_{i}^{L}$ stands for the percentage of the B-scans labeled as L in $X_{i}$, L=AMD, NOR for the Duke dataset. The voting strategy is as follows: if $P_{i}^{\mathrm{AMD}}$ is larger than ε, then the classification result $Y_{i}$ of $X_{i}$ is AMD; otherwise $Y_{i}$ is NOR. How to determine optimal thresholds is important. Some empirical thresholds are given in Refs. 16 and 17. To compare our methods with the voting inference methods using an optimal threshold, we perform tests to demonstrate the relationship between accuracy, sensitivity, specificity, and threshold ε, using fivefold cross-validation for ε=0.1,0.2,…,0.9. The voting classification results are shown in Fig. 4, where the solid lines represent the average values and the shaded part of the corresponding color is the confidence interval. It is evident that ACC achieves the best results when ε=0.6, and we choose the best ACC to compare with our method.

Performance comparisons of our proposed method FTA-ResNet50+CNN_CBAM with other methods are given in Table 2. Table 2 shows that the performance of our proposed method is much better than the others. We note that the voting strategy uses only information that is available at each B-scan from a 3-D OCT volume for classification, whereas our proposed volume-level classifiers can integrate information from B-scans. This maybe the reason that our proposed methods outperform the common voting strategy.

**Table 2:** Performance comparisons with state-of-the-arts on two classes of the Duke dataset (%).

Methods	ACC	SE	SP
Voting strategy	87.76±7.09	90.74±13.25	80.87±18.77
Santos et al.^27^	95.20±2.30	94.20±3.10	97.50±3.20
Qiu et al.^17^	95.88±0.19	98.22±0.72	90.43±2.36
FTA-ResNet50+CNN_CBAM	98.17±0.64	99.26±0.91	95.65±2.75

The method proposed by Sun et al.^29^ achieves a classification accuracy of 94.4% on the Duke dataset, wherein a different train/test set separation is used.

#### 3.5.3 Visualization

In this section, we intuitively demonstrate the reasonableness of the proposed method in reasoning. Taking the 20th volume of an AMD set and the 65th volume of an NOR set and denoting them as AMD20 and NOR65 respectively, in the original Duke dataset as representative samples, these two volumes belong to a training set in an experiment in which D_3 is the test set. We utilized the finetuned ResNet-50 as the B-scan feature extractor.

##### Visualization of B-scan feature vectors

**Fig. 5:**
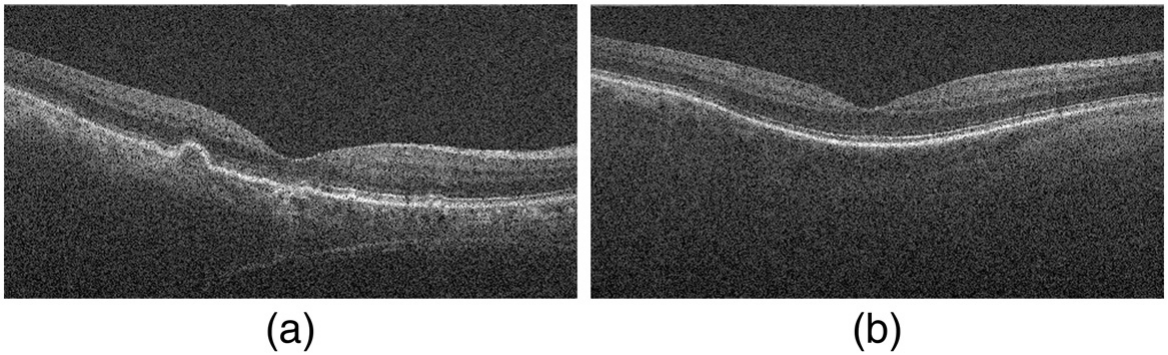
Representative B-scan examples: (a) 50th B-scan in AMD20 and (b) 50th B-scan in NOR65.

**Fig. 6:**
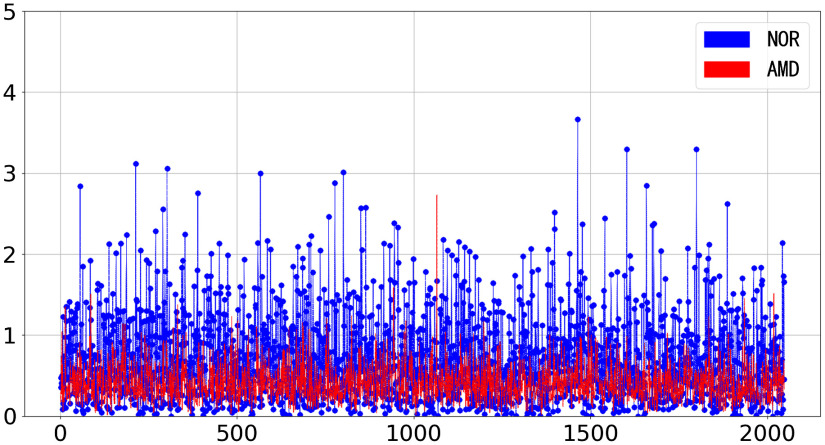
Visualizations of B-scan feature vectors: 50th B-scan (red) in AMD20 and 50th B-scan (blue) in NOR65.

We select a representative AMD B-scan image in AMD20 and an NOR B-scan image in NOR65, as shown in Fig. 5, and visualize their feature vectors in Fig. 6. Figure 6 shows that feature vectors of the AMD and NOR B-scan images have different patterns.

##### Visualization of 2-D feature maps

**Fig. 7:**
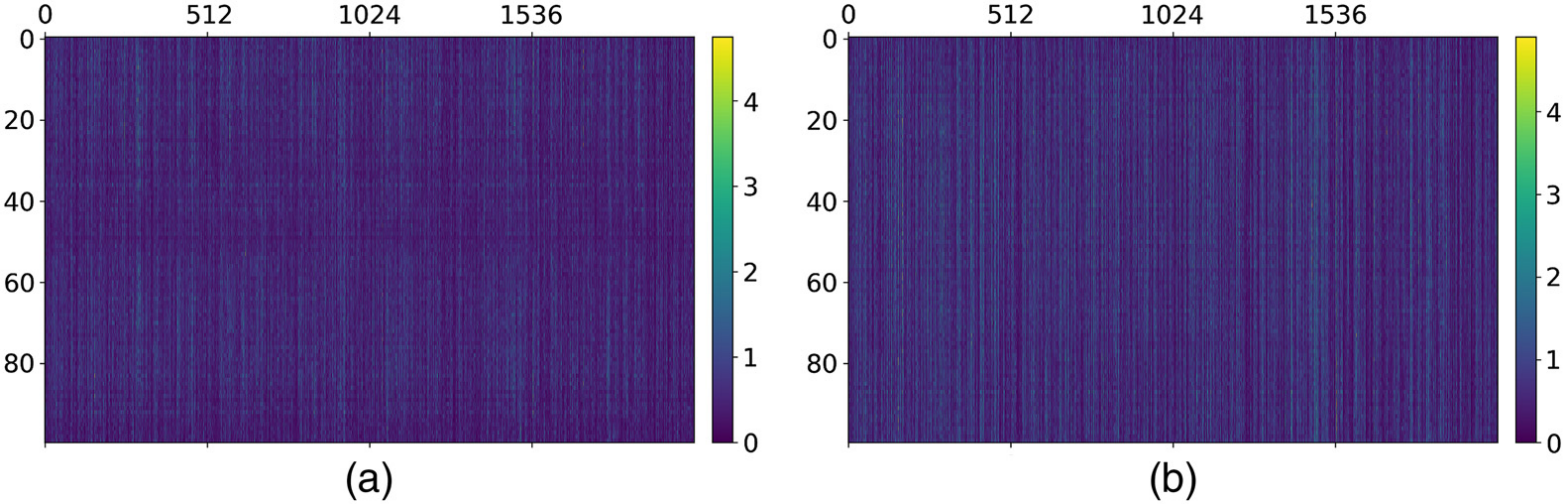
Visualization example of 2-D feature maps (a) AMD20 and (b) NOR65.

**Fig. 8:**
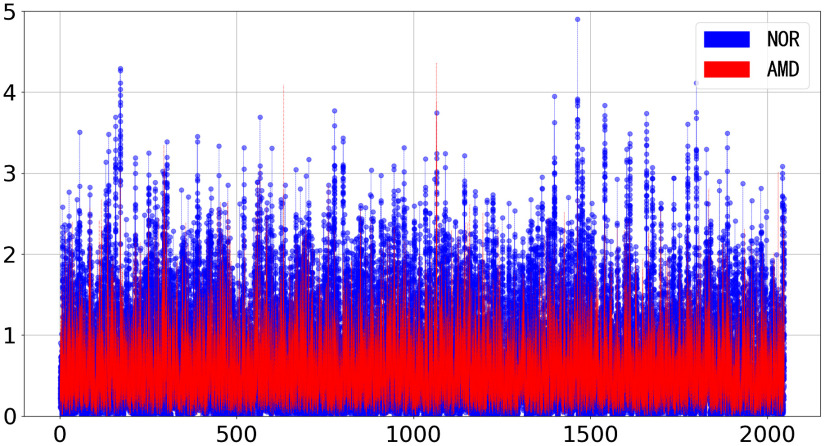
Visualization of the feature vectors of AMD20 and NOR65 from 35 to 65 B-scans.

For AMD20 and NOR65, we visualize their 2-D feature maps with a size of 100×2048, as shown in Fig. 7. In the two maps, their feature values are between 0 and 5, and the color bars show the quantized colors for visualization. Figure 8 visualizes the feature vectors of AMD20 and NOR65 from 35 to 65 B-scans, respectively. Overall, the texture in NOR65 is more uniform than that in AMD20, and NOR65 possesses larger feature values than AMD20 does. The 2-D feature map has data correlation between rows, reflecting the correlation of B-scan features in 3D, and 2-D feature maps of AMD volume and NOR volume are intuitively distinguishable.

### 3.6 Experiments on Tsinghua Dataset

**Fig. 9:**
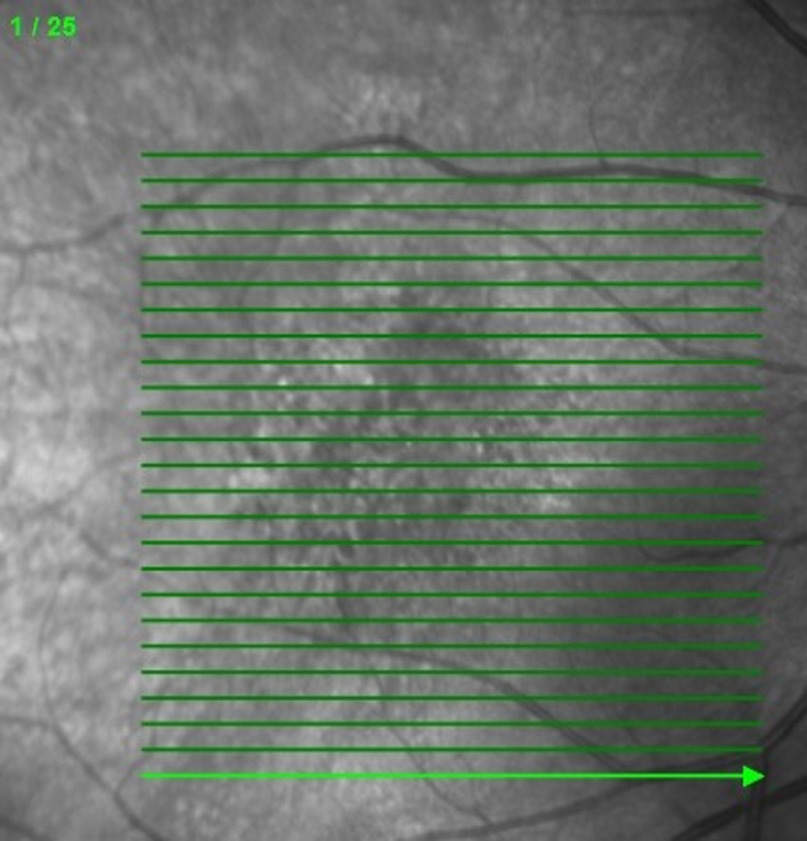
FAST scan mode.

**Fig. 10:**
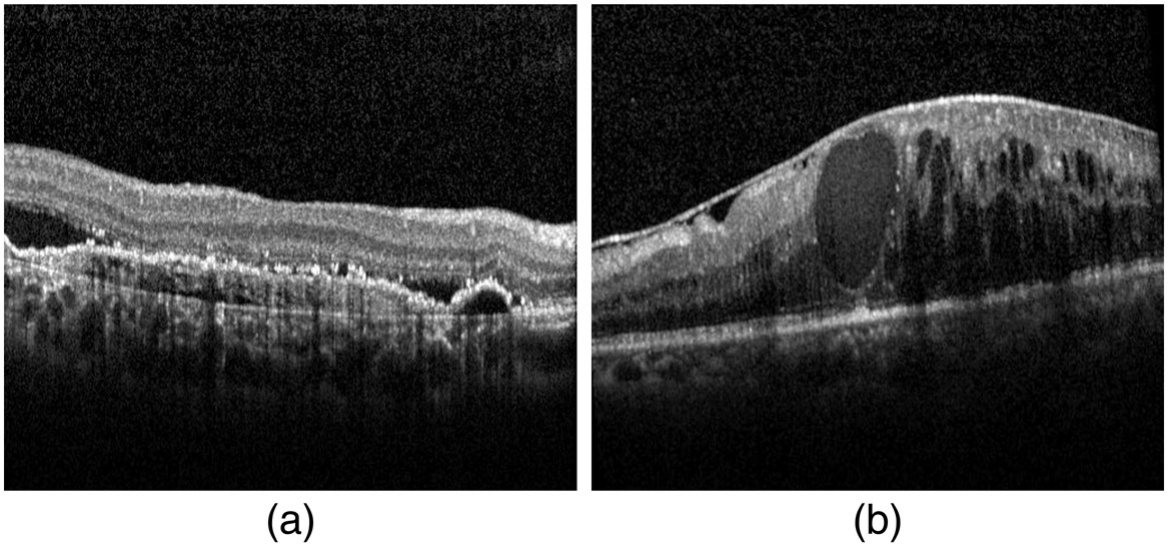
Examples of B-scan images from Tsinghua dataset (a) AMD and (b) DME.

Our private dataset is obtained using FAST scan mode (see Fig. 9), in which every volume has 25 B-scans. These OCT data were collected from patients in clinics, and no volunteers were recruited, so only AMD and DME volumes are included. Two representative examples are shown in Fig. 10. Our observations demonstrate that distinguishing a AMD B-scan from a normal B-scan in the Duke dataset is more difficult than classifying AMD and DME B-scans in the private dataset.

We note that, in general, a dataset often consists of AMD and NOR volumes or DME and NOR volumes. In these cases, NOR volumes are often considered negative samples and AMD or DME volumes as positive samples, and accuracy (ACC), sensitivity (SE), and specificity (SP) are calculated with the equation in Sec. 3.4. For our private dataset, to calculate ACC, SE, and SP using the same equation as before, without loss of generality, we take AMD as a negative and DME as a positive sample.

We test to validate the effectiveness of our proposed algorithms FTA-ResNet50 +SVM, FTA-ResNet50 +CNN, and FTA-ResNet50+CNN_CBAM on classification of AMD and DME volumes. Every volume has 25 B-scans, so the 2-D feature map of the OCT volume is of size 25×2048. For this application, we let n=3 in Fig. 3, i.e., three convolutional blocks are used in our CNN and CNN_CBAM classifiers. We partition the dataset into five parts with roughly the same amount, conduct fivefold cross-validation, and evaluate our proposed methods. Our experiments show that, for the finetuned ResNet50 feature extractors, the classification accuracies (ACC) achieve 100±0.00%, 99.78±0.27%, and 99.89±0.22% for SVM, CNN, and CNN_CBAM volume-level classifiers, respectively. This reveals the effectiveness of the proposed methods on the one hand and implies that it is more distinguishable between AMD and DME on the other hand. To be able to better discriminate the classification performances of SVM, CNN, and CNN_CBAM volume-level classifiers, we redesign our experiment with a different training/test set partition. We randomly select 40% of the AMD and DME volumes as the training set separately, and the remaining 60% of the AMD and DME volumes as the test set. This procedure is repeated five times. The classification results of methods are shown in Table 3.

**Table 3:** Classification performance of our methods on Tsinghua dataset (%), where 40% AMD and DME volumes are training set, AMD is negative, and DME is positive.

Methods	ACC	SE	SP
FTA-ResNet50 +SVM	99.93±0.09	100.00±0.00	99.86±0.18
FTA-ResNet50 +CNN	99.49±0.27	99.78±0.30	99.21±0.42
FTA-ResNet50+CNN_CBAM	99.71±0.25	99.78±0.30	99.64±0.40

Table 3 also shows the effectiveness of our proposed methods. For the private dataset, the volume-level classifier SVM is the best of all, while CNN_CBAM is slightly better than CNN. We conduct further experiments to show the number n of convolutional blocks in the CNN and CNN_CBAM classifiers. Comparing with n=3, the experimental results are a little better for n=2 and a little bit worse for n=4. So n=3 is a feasible number for the private dataset.

### 3.7 Discussion

This report focuses on volume-level classification in which only the label of the OCT volume is known. Our classification scheme was first proposed in Ref. 30. Here, we did an in-depth study. The proposed classification architecture is general, consisting of three modules: B-scan feature extractor, 2-D feature map generation, and volume-level classifiers. The finetuned ResNet-50 is selected as the B-scan feature extractor and the retraining scheme of ResNet-50 is provided. In our finetuning strategy of ResNet-50, the label of an OCT volume is assigned to each B-scan of the volume. An OCT volume with the label AMD often includes many normal B-scans, which would lead to many noisy labels. This kind of disadvantage was pointed out first in Ref. 17, and a relabeling technique was proposed to overcome it. Hence, the finetuning strategy suggested in this paper should be improved by combining it with the relabeling technique or integrating it with attention techniques. Apart from ResNet-50, other classical CNN models could also be considered the backbone networks for B-scan feature extraction. When 2-D feature maps of OCT volumes are generated, how to design classifiers to classify them is another key point. In this aspect, we adopt traditional LSVM and propose CNN with/without attention mechanism as volume-level classifiers. Our experiments show that all of these volume-level classifiers are very successful. Our proposed OCT volume classification methods do not need any OCT denoising or retinal flattening image preprocessing, they outperform the state-of-the-art methods greatly on the publicly available Duke dataset, and they are also very effective on the private dataset. Of course, extending this private dataset to include the NOR data is encouraged and will be a future effort.

In our experiments, finetuning ResNet-50 requires ∼7.1G GPU RAM, whereas training of the SVM and CNN classifiers requires ∼0.6G CPU RAM and 1.8G GPU RAM, respectively. Hence, our classification scheme also saves memory resources, so it is highly suitable for large OCT volume datasets. Therefore, the proposed scheme is very promising in assisting ophthalmologist to screen macular diseases from OCT volume.

For given datasets, our methods were used to recognize macular diseases such as AMD and DME. In principle, the proposed 2-D feature map representation is not limited to OCT volume; it may be adapted to any other 3-D medical data such as volumetric magnetic resonance imaging and/or computed tomography (CT) data.

## 4 Conclusions

We have reported on a general solution for automatic diagnosis of macular diseases using an OCT volume based on its 2-D feature map and CNN with/without attention mechanism.

We describe some implementations of this scheme. Specifically, the finetuned ResNet-50 is used as the B-scan feature extractor to generate a 2-D feature map, and SVM, CNN, and CNN_CBAM are utilized as volume-level classifiers to classify these 2-D feature maps. These classification methods could classify OCT volumes automatically and effectively with high accuracy, and they are potential practical tools for screening of ophthalmic diseases from OCT volume.
